# Three-Year-Olds' Understanding of Desire Reports Is Robust to Conflict

**DOI:** 10.3389/fpsyg.2018.00119

**Published:** 2018-02-19

**Authors:** Kaitlyn Harrigan, Valentine Hacquard, Jeffrey Lidz

**Affiliations:** ^1^Linguistics and Psychology, College of William & Mary, Williamsburg, VA, United States; ^2^Linguistics, University of Maryland, College Park, College Park, MD, United States

**Keywords:** language acquisition, language development, theory of mind, belief, desire, attitude verbs, mental state verbs, linguistics

## Abstract

In this paper, we present two experiments with 3-year-olds, exploring their interpretation of sentences about desires. A mature concept of desire entails that desires may conflict with reality and that different people may have conflicting desires. While previous literature is suggestive, it remains unclear whether young children understand that (a) agents can have counterfactual desires about current states of affairs and (b) agents can have desires that conflict with one's own desires or the desires of others. In this article, we test preschoolers' interpretation of want sentences, in order to better understand their ability to represent conflicting desires, and to interpret sentences reporting these desires. In the first experiment, we use a truth-value judgment task (TVJT) to assess 3-year-olds' understanding of want sentences when the subject of the sentence has a desire that conflicts with reality. In the second experiment, we use a game task to induce desires in the child that conflict with the desires of a competitor, and assess their understanding of sentences describing these desires. In both experiments, we find that 3-year-olds successfully interpret want sentences, suggesting that their ability to represent conflicting desires is adult-like at this age. Given that 3-year-olds generally display difficulty attributing beliefs to others that conflict with reality or with the child's own beliefs, these findings may further cast some doubt on the view that children's persistent difficulty with belief (think) is caused by these kinds of conflicts.

## Introduction

Human beings explain each other's behavior in terms of concepts like belief and desire. When we see, say, that Sally opened the cupboard, we infer that it was because she *believed* there was food inside and she *wanted* to eat. An important question in the domain of cognitive development centers around the origins of these concepts. When do children understand that other people have beliefs and desires?

There has been considerable controversy concerning the age at which children can be said to be sensitive to other people's beliefs (Johnson and Maratsos, 1977; Wimmer and Perner, 1983; de Villiers, 1995, 2005, 2007; de Villiers and de Villiers, 2000; Wellman et al., 2001; de Villiers and Pyers, 2002; Perner et al., 2003; Lewis, 2013; Lewis et al., 2017, and others). This question is somewhat vexed by the fact that different researchers set the standard for what counts as good evidence for belief attribution differently. All are agreed that the attribution of false beliefs to someone else is required to demonstrate mastery of the belief concept. However, they disagree about what counts as evidence of false belief attribution, with some arguing that implicit measures indicate that the concept is in place early (Onishi and Baillargeon, 2005; Southgate et al., 2007; Baillargeon et al., 2010; Kovács et al., 2010), and others arguing that such measures may be reflective of heuristics or concepts with similar extensions, and that only explicit verbal measures count as definitive evidence for belief understanding (Apperly and Butterfill, 2009; Thoermer et al., 2012; Butterfill and Apperly, 2013; Heyes, 2014). But certainly all are in agreement that when children pass explicit, verbal false belief tasks, the relevant concept must be in place. The gold standard for demonstrating the full richness of belief is the use and comprehension of the words that make reference to that concept.

Likewise, we can ask what the behavioral entailments associated with an adult-like desire concept are. And again we find the same kinds of issues. A desire concept should support an ability to represent desires that are counterfactual (i.e., *conflict* with reality), that conflict with one's own desires or with those of others. It should support a representation of desires about states of affairs, not just about objects, and an adult-like comprehension of words like *want* should depend on having this concept.

Prior work using implicit measures suggests that children are sensitive to the goals of human agents from as young as 5 months of age (Woodward, 1998, 1999, 2003), and that they are sensitive to the goals of others which differ from their own by 18 months of age (Repacholi and Gopnik, 1997). However, it is not clear that success on these tasks requires a full-fledged concept of desire: perhaps young children grasp that others have desires that differ from their own, so long as these desires do not conflict with each other; perhaps children make sense of intentional actions merely teleologically, i.e., in terms of objective facts and goals (Perner and Roessler, 2010); perhaps children have an “objective” notion of desire about what is generally good, but not yet a desire concept that holds across counterfactual, subjective and conflicting desires (Perner et al., 2005). While a few studies explicitly test children's understanding of such a “subjective” concept of desire (Moore et al., 1995; Rakoczy et al., 2007; Rakoczy, 2010), these studies report considerable variability in children's performance, both within and across tasks.

In this paper, we probe young children's concept of desire through their comprehension of desire reports using the verb *want*. We present two experiments testing whether 3-year-olds can understand sentences reporting desires under three conditions: (1) counterfactual desires that conflict with reality; (2) desires that conflict with another person's desires; and (3) desires that conflict with the child's own. We show that even with such tests, children are able to understand *want* before their fourth birthday. This suggests that 3-year-olds have a robust adult-like understanding of *want*, which is not affected by additional conflicts. Three-year-olds' success with this diversity of desire sentences implies that they have a robust understanding of the desire concept, and can represent even conflicting desires.

## Previous research

Many previous studies have explored children's understanding of the desire concept and the verb *want*. These studies have focused on desires for objects, conflicting desires for objects, desires for states of affairs, and conflicting desires for states of affairs.

Several studies have examined whether children expect an agent to act in accordance with her desires. Wellman and Woolley (1990) asked 2-year-olds to make predictions about various characters' actions, based on their desires. They found that 2-year-olds were successful at predicting actions related to people's simple desires. Tasks using eye gaze measures have been used to test even younger children's desire representations. Woodward (1998) tested 5- and 9-month-olds in a looking-time paradigm. In her task, infants saw an agent reach for one of two objects situated next to each other. After children were habituated to this action, the positions of the objects were switched, and the agent either reached for the same toy (now in a new position), or made the same arm movement (same direction, but now reaching for a new toy). Infants looked longer when the agent reached for a new toy, even though the agent's arm motion was the same as in familiarization. They did not look longer when a non-human grasping device was used, implying that only the movement of the hand reflected the desires of an agent. This pattern held for both 9-month olds and even 5-month olds (although with weaker results). This set of studies shows that by as young as 5 months old, infants can encode the actions of agents, attribute goals to them, and expect people to act in accordance with those goals. However, we might view these results as reflecting an understanding of goals and not desires *per se*.

Other tasks look at slightly older children's ability to represent conflicting desires for objects. Repacholi and Gopnik (1997) examined whether 14- and 18-month-olds could appropriately represent the desires of an agent that potentially differed from the child's own. In the task, the child was introduced to two familiar and distinctive foods: goldfish crackers and broccoli. After introducing each food, the researcher produced a salient response—either positive or negative—toward each of the foods, and then requested that the child give her some food. To control for the possibility that children assumed that everyone has the same desires, or the possibility that the child would give the experimenter their own non-desired food in order to keep the desired food for themselves, the researchers manipulated whether the experimenter expressed preference for the same or the opposite food from the child's preferred food. The authors found that most of the children preferred the crackers and that at 14-months, children were more likely to give the researcher the crackers, regardless of which food she had expressed preference for. By 18 months, however, they were more likely to give the experimenter the food that she preferred, regardless of whether it matched the child's preference or not. This study shows that by 18 months, children seem to understand that different people may have desires that differ from their own. As Rakoczy et al. (2007) point out, however, while the experimenter's desire for broccoli differs from the child's desire for crackers, the two desires are not incompatible. Perhaps children's notion of desire that allows them to pass the task is a mere “objective” desire about what is generally good: broccoli for the experimenter, and crackers for the child (This would be in line with the teleological account of Perner and Roessler, 2010). Hence, the task shows at least that children know that different objects may be good for different people and possibly that children understand that others can have desires that differ from their own. However, it does not show that children understand *conflicting* desires.

To address conflicting desires, researchers have probed children's understanding of desires about states of affairs, as reported by *want* sentences. Here the results are mixed. Perner et al. (2003) tested children's interpretation of *want* sentences in German-speaking children, and compared it to their understanding of *think* sentences. Children (2.5–4.5) saw six stories, each of which was accompanied by a drawing. For example, in one story, Mom and Dad were in one room and their son Andy was watching television in his bedroom. In the *want* condition, Mom asked Dad to see what Andy was doing. Dad asked Mom what Andy should do, and Mom answered, “Andy should go to bed.” Then the child was asked the *want* test question, shown in (1).

(1) *Was will die Mutter, dass Andreas tut?*
what wants the Mom, that Andy does
‘*What does Mom want Andy to do?’*

In the *think* condition, Dad asked Mom what Andy was doing and she answered, “Andy is going to bed.” Then the child was asked the *think* test question, shown in (2).

(2) *Was glaubt*^1^ *die Mutter, dass Andreas tut?*
what believes the Mom, that Andy does
‘*What does Mom believe that Andy is doing?’*

They found that children gave more correct answers to the questions with *want* than those with *think*, and concluded that it is easier for children to remember discrepant desires than discrepant beliefs.

However, it is not so clear that the mother's desire in the story is in conflict with reality. Note that English and German differ in the syntactic properties of the verbs *think* and *want*. In English, *think* takes a tensed complement, while *want* obligatorily takes an untensed complement. By default, the untensed complement of *want* receives a future-orientation when the verb is eventive [as in (1)]. The temporal interpretation of the complement of *think* depends on the tense in the complement: with a present tense, the belief is present-oriented (a future-orientation would require a future tense morpheme, as in ‘*Mom thinks that Andy WILL go to bed*). In German, both *think* and *want* take tensed complements^2^. However, with *want* (but not with *think*), it is nonetheless possible to get a future-orientation with a present tense in the complement (in fact it is the preferred interpretation)^3^. Sentence (1) can thus be interpreted in two ways. It can get the interpretation in (3), which sets up a conflict between desire and reality, but it also allows the (preferred) interpretation in (4), which is future-oriented and thus avoids a conflict with reality.

(3) *What does Mom want Andy to be doing (right now)?*
(4) *What does Mom want Andy to do (later)?*

If children interpret (1) as meaning (4), Mom's desire can still be satisfied if Andy's future actions match her current desire, in which case there is no conflict between her desire and reality. Thus, this task did not require children to interpret *want* under truly conflicting conditions.

A pilot study reported in de Villiers (2005) controls better for the possibility of future-oriented readings by using *want* sentences with a participial complement. This structure forces the time of the event to overlap with the time of the desire, making this a better test of an actual present-oriented conflict. In a representative story, a character named Bella was painting, but her mother thought that she was playing on the computer and was happy about it. Children were asked the question in (5).

(5) *Does Mom want Bella playing on the computer?*

De Villiers found that children were successful in interpreting these *want* sentences. While these results are suggestive, the number of children tested was small, and each only got two critical trials. Thus, a larger study is necessary to assess children's comprehension of *want* sentences reporting counterfactual desires. Furthermore, while the participial complement does not allow a future orientation, it is not a fully sentential complement. This may make interpretation of sentences like (5) easier, and these results may consequently overestimate children's knowledge of *want*.

To see if children truly understand that agents can have conflicting desires and do not merely rely on a merely “objective” concept of desirability for states of affairs, rather than on a mental representation of desires, Rakoczy et al. (2007) tested whether children fail to correctly interpret *want* in cases where desires are subjective, with different people having *non-compatible* desires (using a task first introduced by Lichterman, 1991). They showed children (3.0–3.6) stories in which two characters quarreled about which of two either compatible or incompatible outcomes they preferred. In the compatible desires stories, two characters, Tom and Susi, were each in boats. Tom wanted his boat to go to one location, Susi wants her boat to go to another location. The boats then go to one of the two locations. The incompatible desires stories are the same, except that both characters were in the same boat together, thus it was impossible for each character's desire to be satisfied simultaneously. After the story, the children were asked the test questions shown in (6) and (7).

(6) *Susi wanted the boat to go where?*
(7) *And Tom wanted the boat to go where?*

Children succeeded on this task, suggesting that they can both represent incompatible desires and interpret *want* sentences before their fourth birthday. Note however that the future orientation of (6) and (7) could still prevent a conflict between reality and the desire, and hence not provide a stringent test of children's ability to represent incompatible desires. The question in (6) describes a past desire about an outcome future to this past desire time. Although the boat did go to one of the two locations at a time future to this desire time (namely, at the end of the story), the future is open, and it is possible that the boat could still subsequently go to a second location, and thus satisfy the desire in the near future. To rule out this possibility, it is necessary to make explicit that the desire is about a *concurrent* state of affairs.

Finally, two studies probe whether children understand that an agent can have desires that conflict with their own desires. Moore et al. (1995) looked at 3-year-olds' understanding of conflicting desires in a task in which they played a game against a puppet, “Fat Cat.” The child and Fat Cat each had to solve their own jigsaw puzzle for which they needed parts from a blue or a red box. In each round, a card was drawn from a stack, turned around and shown to be either blue or red. Both players could then take a piece from the corresponding box. At first, both players needed pieces from the same box (e.g., red box). However, there came a point where their needs diverged, and thus their desires for which color the card should be became incompatible (still red for Fat Cat, blue for the child). At this point the child was asked three control questions and two test questions below [(8) is about the other's desire, (9) about the child's own outdated desire]:

(8) *Which color card does Fat Cat want now?*
(9) *Which color card did you want last time?*

Only 7 of 20 children passed both test questions on the conflicting-desire task, leading Moore et al. to conclude that when children are forced to represent incompatible and conflicting desires, they have difficulty interpreting sentences with *want*.

Rakoczy et al. (2007) and Rakoczy (2010) were concerned that the methodology used in the Moore et al. study may have underestimated children's knowledge, as the task was very complex. Using a simpler game format modeled on the Moore et al. study, they tested both conflicting “third person desires,” where two puppets played against each other, and conflicting “first person desires,” where the child played against a puppet. In this task, children (3.0–3.6) worked together with a puppet to make a sticker book, but only one sticker could go inside. A “chance machine,” out of which a marble was dispensed, determined one of two sticker possibilities: one was an exciting sticker, and one was a boring sticker. Children always preferred the more exciting sticker, and the puppet expressed interest in the other sticker. The children were asked the test questions shown in (10, 11).

(10) *You want the marble to roll where?*
(11) *Rudi* [puppet] *wants the marble to roll where?*

Rakoczy et al. found that the simpler probe of conflicting desires did improve performance, compared to the original Moore et al. study, with accuracy around 55% overall. There were no differences between first and third person conditions. However, children's performance was still not adult-like. There may, however, be further methodological concerns in this improved task. As in the Moore et al. study, there were only one or two critical trials. Additionally, the game was still fairly complex for preschoolers, and the authors did not report training or set a criterion to determine whether children understood the rules of the game. Thus, they potentially underestimated children's understanding of *want*, as failures may have been due to a lack of understanding of the rules of the game. To get at children's understanding of *want* in conflicting situations, it is critical to exclude children who do not understand these rules, to ensure that errors are due to difficulty processing or understanding conflicting desires, and not to confusion about the game. To sum up, it remains an open question whether 3-year-olds can understand *want* sentences (and the underlying desire concept), in cases of conflicting desires.

The previous tasks looking at children's interpretation of *want* can be improved in several ways, in order to license better inferences about children's knowledge. The first is in the temporal orientation of the attitude verb. As discussed above, *want* sentences often get a future-oriented reading, and thus do not necessarily report a desire that *conflicts* with reality, or with another person's desire. Experiment 1 controls for the temporal orientation of the complement of *want*, allowing us to test children's understanding of *want* when there is a conflict between the reported desire and reality. Experiment 2 tests children's understanding of *want* sentences used to report desires that conflict with their own. We improve on the methodology of the previous studies by including a thorough training in those aspects of the task that do not have to do with desire, and excluding participants who do not understand the rules of the game. This improvement decreases the likelihood that experimental artifacts will lead us to underestimate children's understanding of *want* sentences.

## Experiment 1: conflict with reality

Experiment 1 tests *want* sentences that force a present-orientation, and thus describe desires that potentially conflict with reality and with another character's desires.

### Subjects

Participants were 44 children aged 3.0–4.0 (mean = 3.8). Sixteen additional children were excluded from the task, either due to *yes-* or *no-*biased responses, or parental interference. Children in all three studies were recruited from the College Park, Maryland area, and were reported by their parents to be monolingual speakers of English. Participants were recruited via telephone or email from the University of Maryland Infant Studies Database.

### Design and materials

Experiment 1 was a *Truth Value Judgment Task* (TVJT), which requires children to evaluate and potentially correct sentences uttered by a “silly” puppet (Crain and McKee, 1985; Crain and Thornton, 1998). TVJT tasks gauge whether children at a given age pair certain linguistic stimuli to a given situation in an adult-like way, or whether their interpretation of the stimuli differs in some way from adult judgments. In this task, children listened to stories with pictures. They were told that a puppet who was “very silly and sometimes gets things wrong” was listening to the stories as well, and asked to tell the puppet whether he was right or wrong after every utterance. Each child saw eight stories. After each story the puppet uttered two sentences: a filler sentence and a test sentence. The fillers were intended to ensure that the child was paying attention and had a basic understanding of what happened in the story (e.g., *Megan is at the grocery store with her {Mom/Dad}*). Test sentences had a sentential complement which forced a present orientation by using a progressive (“be ___ING”), and the temporal modifier “right now” [see (12)].

(12) *Mom wants Megan to be sitting in the grocery cart right now*.

There were a total of eight stories, each with two different versions. Between subjects we manipulated whether the stories contained a desire that conflicted with reality (conflict condition) or not (no conflict condition). Each of the stories described a situation in which a child starts out doing a given activity, and then an adult asks the child to either continue doing the same activity (stay condition) or switch to a new activity (switch condition). This manipulation was within subjects. Half of the conflict stories were stay stories, the other half were switch stories. Each story was used in both conflict and no conflict conditions, and each story was used in both stay and switch conditions. This ensured that each story was equally plausible as a conflict or no conflict situation, as well as a switch or stay scenario. Additionally, it ensured that in both the conflict and no conflict conditions, the character did not always start and end doing the same activity. We also manipulated the truth-value of the test sentences within subjects. Table 1 illustrates the within- and between-subjects factors in Experiment 1.

**Table 1:** Within and between subjects factors in Experiment 1.

**conflict/no conflict (between subjects)**	**switch/stay (within subjects)**	**truth (within subjects)**
conflict	switch	*True*
		*False*
conflict	stay	*True*
		*False*
no conflict	switch	*True*
		*False*
no conflict	stay	*True*
		*False*

#### Sample story

**Figure 1:**
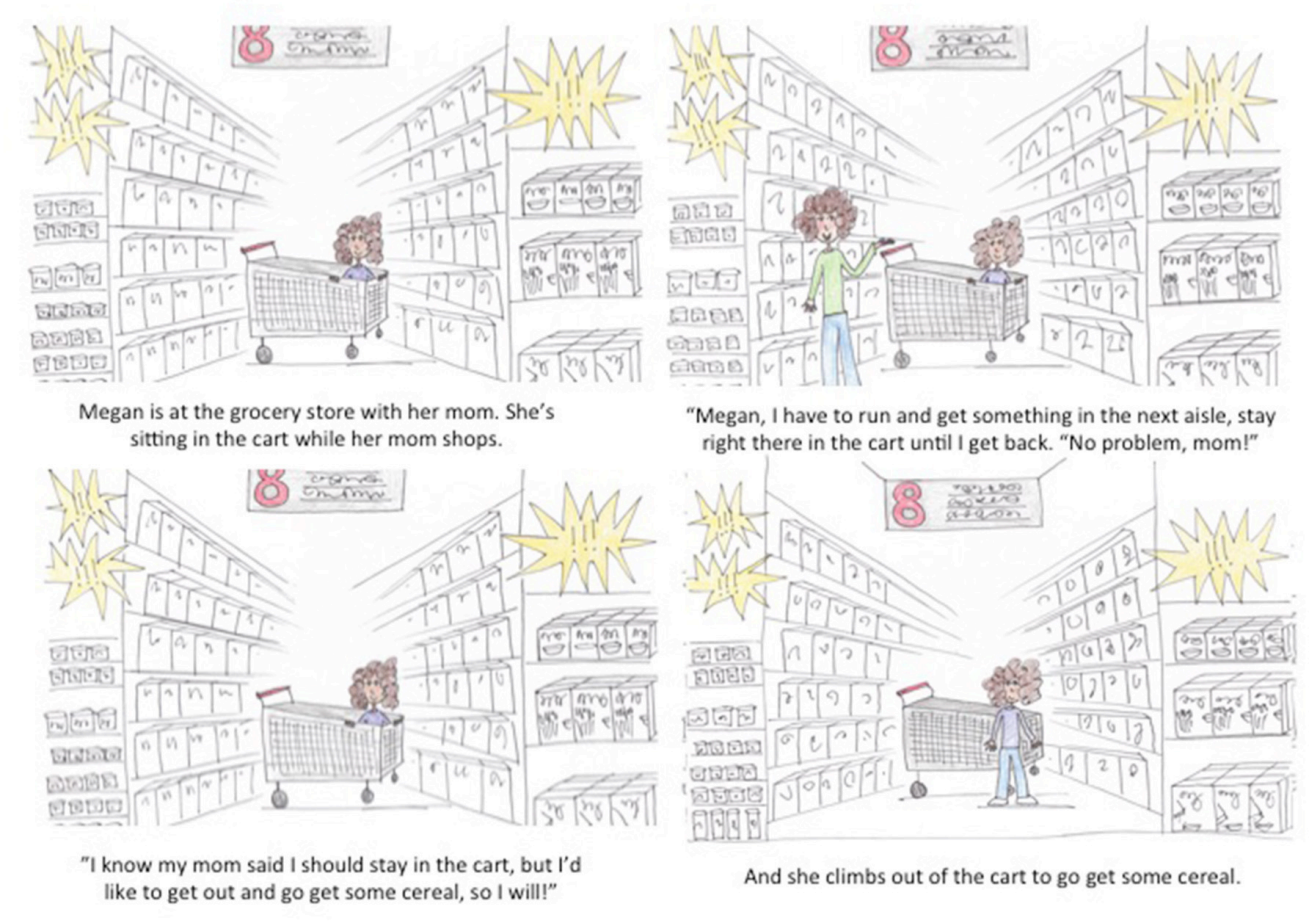
Experiment 1 sample story.

The stories consisted of four pictures each. Each picture represented about one sentence of a story. A sample of the text of one story is laid out in Table 2 and Figure 1 (see Appendix for complete set of stimuli).

**Table 2:** Experiment 1 sample story.

Introduction phase	*Megan is at the grocery store with her mom. She's sitting in the cart while her mom shops*.	
stay/switch phase	stay: *Megan's mom says, “Megan, I have to run and get something in the next aisle, stay right there in the cart until I get back. And Megan says, “No problem, mom!”*	switch: *Megan's mom says, “Megan, I have to run and get something in the next aisle, can you climb out of the cart and go get some cereal? And Megan says, “No problem, mom!”*
conflict/no conflict phase	stay/conflict: *Mom leaves, and Megan says to herself, “I know my mom said I should stay in the cart, but I'd like to get out and go get some cereal, so I will!”*	switch/no conflict: *Mom leaves, and Megan says to herself, “I'd like to stay right here in the cart, but my mom said to get out of the cart and go get some cereal, so I will!”*
	stay/no conflict: *Mom leaves, and Megan says to herself, “I'd like to get out of the cart and go get some cereal, but my mom said to stay in the cart, so I will!”*	switch/conflict: *Mom leaves, and Megan says to herself, “I know my mom said to get out of the cart and go get some cereal, but I'd like to stay right here in the cart, so I will!”*
Outcome phase	stay/conflict: *So she climbs out of the cart to go get some cereal*.	switch/no conflict: *So she climbs out of the cart to go get some cereal*.
	stay/no conflict: *So she stays right there in the cart*.	switch/conflict: *So she stays right there in the cart*.
Test sentence	stay/conflict	switch/no conflict
true	*Mom wants Megan to be sitting in the cart right now!*	*Mom wants Megan to be getting cereal right now!*
false	*Mom wants Megan to be getting cereal right now!*	*Mom wants Megan to be sitting in the cart right now!*
	stay/no conflict	switch/conflict
true	*Mom wants Megan to be sitting in the cart right now!*	*Mom wants Megan to be getting cereal right now!*
false	*Mom wants Megan to be getting cereal right now!*	*Mom wants Megan to be sitting in the cart right now!*

#### Procedure

Each child was tested in a quiet room with two experimenters. One experimenter told the child the stories and showed her the pictures, while a second experimenter controlled the puppet and uttered the filler and test sentences. The second experimenter also coded the child's responses. Permission was obtained from parents to video record each subject for an additional round of coding off-line.

The experiment began with the child being introduced to a silly puppet, “Froggy.” The experimenters were somewhat flexible with the script, adjusting to each child's level of attention, but followed the following script fairly closely:

“*We're going to be looking at some pictures and hearing some stories that go along with them. And our friend Froggy is going to listen to the stories with us, ok? And after we hear a story, Froggy's gonna try to tell us what happened in the story. But sometimes, he's not a very good listener. And so sometimes when he tells us, he might get it wrong, ok? And you get to tell us whether Froggy was right or wrong. Does that sound like a good plan? OK, so listen carefully, because he says silly stuff sometimes!”*

The child then practiced interacting with Froggy. First Froggy named a few items, and the child practiced telling him *yes* and *no*. The child was corrected during this practice phase if they did not correctly tell Froggy *yes* and *no*. Then the child was told two very simple stories, and practiced responding to sentences Froggy said about the stories. Froggy was correct once and incorrect once. Again, the child was corrected if she did not respond correctly to Froggy's sentences.

During each test trial, Experimenter 1 read the story and showed the child the pictures. After each story, Experimenter 1 turned to Froggy and asked “*What happened in that story, Froggy?*” Then Froggy uttered the filler sentence, after which the experimenter turned to the child and asked “*Did Froggy get it right?*.” Then the child either responded *yes* or *no*. After the filler, Froggy uttered the test sentence, and the child was again asked whether or not he was right and given the chance to respond *yes* or *no*. Experimenter 1 gave feedback to Froggy that was in accordance with how the child had responded—“*Good job, Froggy!*” when the child said that Froggy was correct, and—“*Oh, silly Froggy! Try again next time!*” when the child said that Froggy was incorrect. The entire experiment took around 8–10 min per child.

### Results

#### Coding

Children's responses were coded online by the second experimenter. Four out of the 60 videos were coded by a second experimenter offline, because coding did not happen online. Responses were coded as *yes*, or *no*. One response (out of 1,080 total responses) was unintelligible. An additional 25% (11 videos) were coded offline by an additional coder. We found 99.4% agreement between coders (Cohen's Kappa = 0.989).

#### Filler accuracy

The fillers were designed to ensure that children were listening to the story. They did not rely on understanding *want*. Children who answered either all *yes* or all *no* to 15 out of 16 total items (8 fillers, 8 test sentences) were excluded from analysis (the average age of the excluded children was 3.4, which was not significantly different from the age of the included children). Twelve children were excluded due to *yes-*biased responses (20%). Three children were excluded due to *no-*biased responses (5%). One additional child was excluded due to parental interference. The age range that we were testing for this study is quite young for the TVJT paradigm, which likely contributed to the high number of children with *yes-* or *no-biases*. Overall filler accuracy, including children who were excluded, was 67%. Filler accuracy for the children included in the analysis was 80%.

#### Truth-value judgments

The results for each condition are shown in Table 3.

**Table 3:** Percent yes responses by condition for Experiment 1.

**conflict/no conflict (between subjects)**	**switch/stay (within subjects)**	**truth (within subjects)**	**% Yes responses children 3.0–4.0**
conflict	switch	*True*	93.2
		*False*	13.6
conflict	stay	*True*	79.1
		*False*	17.8
no conflict	switch	*True*	79.5
		*False*	11.4
no conflict	stay	*True*	93.2
		*False*	15.9

We used mixed-effect logit models (Bates and Sarkar, 2007) to analyze the results. These models are well suited for analyzing categorical data (Baayen, 2007; Jaeger, 2008). The reported models have random intercepts. These models predict the probability of a specific response (a correct answer) in the different conditions (see Agresti, 2002; Jaeger, 2008). We ran a mixed-effect logit model with “yes” responses as the dependent measure, with Conflict, Switch, and Truth as fixed effects, and Item and Subject as random effects. We tested for each of the two-way interactions, testing Truth against a model dropping each of the fixed effects. We find a main effect of Truth [${\text{X}}_{\left(1\right)}^{2}$ = 6.41, *p* < 0.001], but no main effects of Conflict [${\text{X}}_{\left(1\right)}^{2}$ = −0.67, *p* = 0.504] or Switch [${\text{X}}_{\left(1\right)}^{2}$ = −1.82, *p* = 0.068]. We find a significant two-way interaction between Conflict and Switch [${\text{X}}_{\left(1\right)}^{2}$ = 1.99, *p* = 0.047], but no other significant two-way interactions. We then tested the full model against a model with only Truth and the interaction between Conflict and Switch as fixed variables. We find a significant main effect of Truth [${\text{X}}_{\left(1\right)}^{2}$ = 9.65, *p* < 0.001], but no main effects of Conflict [${\text{X}}_{\left(1\right)}^{2}$ = −1.05, *p* = 0.29] or Switch [${\text{X}}_{\left(1\right)}^{2}$ = −1.79, *p* = 0.073], and a marginally significant interaction of Conflict and Switch [${\text{X}}_{\left(1\right)}^{2}$ = 1.95, *p* = 0.051]. The main predictor of children's “yes” responses was Truth. The best-fit model was one in which only Truth and the interaction between Conflict and Switch predicted children's “yes” responses, with the effect of Truth being much greater than that of the Conflict/Switch interaction^4^. Children were significantly more likely to respond *yes* to the True items, and *no* to the False items, regardless of whether the item was a conflict or a no conflict item, or whether it was a switch or stay item. These results indicate that 3-years-olds behave as if they understand *want* sentences correctly, even when they are present-oriented and describe a desire that conflicts with reality^5^.

### Experiment 1 discussion

In this study, we set up situations in which characters had desires that conflicted both with reality, as well as with another character's desires. We used linguistic stimuli that disallowed future-oriented readings of *want* sentences to ensure that the reported desires were about concurrent states of affairs, and conflicted with reality and across characters. We find that 3-year-old children are fully adult-like in interpreting such *want* sentences. This shows that 3-year-olds can correctly understand *want* sentences used to describe counterfactual desires^6^.

## Experiment 2

Previous results looking at 3-year-olds' ability to understand reports of desires that conflict with their own are inconsistent, and raise several methodological concerns. Experiment 2 remedies these concerns, by ensuring that children fully understand the rules of the game before they were tested on *want*, and by including more critical trials. We set up a task where children play a game with a puppet, in which their desires sometimes conflict, and then children are asked about those conflicting desires. This task requires children to maintain in memory both their own desires and the puppet's desires.

### Subjects

Participants were 43 children aged 3.0–4.0 (mean = 3.8). Twelve additional children were excluded from the task: 10 did not pass the practice, and two did not finish the task.

### Design and materials

Experiment 2 was set up like a game. The child played with a puppet, *Froggy*, while another puppet, *Booboo*, was “learning” and said things about the game. The child's job was to tell Booboo whether he was right or wrong. The experimenter flipped colored cards (four different colors), and depending on the color of the card, the outcome was either (1) positive for Froggy, (2) the child, (3) both of them, or (4) neither of them (the positive outcome was that someone got to stamp their paper). This set-up induced desires in the child, which sometimes conflicted with the puppet's. The child was told that *Booboo* was “not very good at colors and sometimes gets things mixed up,” and was asked to tell Booboo whether he was right or wrong after every utterance. Each child participated in 4, 8, or 12 practice trials and sixteen test trials. The purpose of the practice trials was to teach the child how the game is played, and to have a measure to exclude children who did not understand how the game worked. The practice trials involved Booboo uttering a sentence that the child had to correct, just like the test trials, but the sentences were about the structure of the game, not a desire. An example of a practice question is shown in (13).

(13) *Oh, I see how the game works! When it's green, Froggy gets to stamp!*

After the child corrected Booboo, the experimenter flipped the card, and asked the child to tell everyone who got to stamp based on the color of the card. This ensured that participants understood the rules of the game and were comfortable playing before the test trials started. Each child had at least four and at most 12 practice trials. We continued with the practice until the child got four in a row correct, and then we moved on to the test trials. If the child did 12 practice items and did not learn how the game worked, they did not move on to the test trials and hence were not included in the analysis.

Each test trial consisted of two test sentences [examples in (14) and (15)], one about Froggy's desire and one about the child's desire.

(14) *Froggy wants the card to be green!*
(15) *You want the card to be green!*

After Booboo uttered the test sentences and the child said whether he was right or wrong, the experimenter flipped the card to reveal who really would get to stamp. The experimenter then asked the child the filler question, which was about the outcome based on color [example in (16)], and then the appropriate player(s) stamped their paper(s).

(16) *Oh! We got green! Who gets to stamp when we get a green card?*

The fillers were intended to ensure that the child was paying attention and understood how the game worked. Children were encouraged to try again if they got the fillers incorrect. This happened very rarely during the game.

This study was a 2 × 2 × 2 design, and all manipulations were within subjects. We manipulated whether we were asking about a desire with a conflict (conflict condition) or not (no conflict condition). Additionally, we manipulated whether we were asking about a positive outcome from the child's perspective (positive condition) or not (negative condition). We also manipulated whose desire we were asking about (sentence), the child's (child desire condition) or Froggy's (Froggy desire condition). The test sentence is *true* when it reports a positive outcome for its subject, and *false* when it reports a negative outcome for its subject, except in the condition where both the child and Froggy get to stamp, in which case the sentence can be judged as either true or false: the outcome where both players get to stamp may be judged as undesirable, depending on how competitive one takes the game. We counterbalanced order between subjects. Table 4 illustrates the within-subjects factors in Experiment 2.

**Table 4:** Within-subjects factors in Experiment 2.

**conflict/no conflict**	**positive/negative**	**sentence (Froggy vs. Child desire)**	**truth**
conflict	positive (child stamps)	“froggy wants…”	False
		“you want…”	True
conflict	negative (Froggy stamps)	“froggy wants…”	True
		“you want…”	False
no conflict	positive (Child & Froggy stamp)	“froggy wants…”	True/False
		“you want…”	True/False
no conflict	negative (No one stamps)	“froggy wants…”	False
		“you want…”	False

Color and outcome were counterbalanced within subjects, so that every color and every outcome occurred an equal number of times during the game. We also rotated which colors were paired with which outcomes throughout the game, to ensure that a color bias would not affect the results. We rotated a total of four times during the game, after every four sets of test questions. Within each of the four blocks, each color and each outcome occurred one time. A schematic of a trial is shown in Table 5.

**Table 5:** Sample of trial in Experiment 2.

**Booboo:** Froggy wants the card to be blue!
**E1:** Did Booboo get it right?
**Child:** yes/no
**E1:** Good job/try again, Booboo!
**Booboo:** You want the card to be green!
**E1:** Did Booboo get it right?
**Child:** yes/no
**E1:** Good job/try again, Booboo! OK, let's flip! [**E1 flips card**] Oh! we got green! What happens when we get a green card?
**Child:** ____ gets to stamp!
**E1:** Good job! Let's stamp! … ok, Booboo, tell us something about the game …

#### Procedure

Experiment 2 began with the child being introduced to “Froggy,” with whom they would be playing the game. The experimenters were somewhat flexible with the script, adjusting to each child's level of attention, but the experimenters followed the following script fairly closely:

“*We're going to play a game with Froggy today where we get to flip cards! And every time that we flip a card, someone gets to put a stamp on their paper. Froggy loves stamps… do you like stamps? OK, so every card that we flip has a color, and we can look at the board [experimenter points] to see who gets to stamp when we flip that color. OK, so when we flip a green card, you and Froggy both get to stamp. When we flip a brown*^7^ *card just Froggy gets to stamp. When we flip a blue card just you get to stamp. And when we flip a pink card no one gets to stamp.”*

Then the child was introduced to the silly puppet, Booboo:

“*OK, one more thing! Froggy's friend Booboo the baboon wants to learn how to play the game, so he's going to watch us play. But he's not very good at colors, so sometimes he gets things mixed up! Sometimes he's going to try to tell us something about how the game works, but he might get it wrong, and your job is going to be to help him out and tell him whether he's right or wrong so he can learn how to play the game. How does that sound?”*

The child then practiced interacting with Booboo. First Booboo practiced naming colors, half of which he got right and half of which he got wrong, and the child practiced telling him *yes* and *no*. The child was corrected during this color practice phase if they did not correctly tell Booboo *yes* and *no*. Then we moved on to the practice phase, where the child saw between 4 and 12 practice trials. Again, during this phase the child was corrected when they made an error.

After sufficient practice, we moved on to the test phase. During each test trial, Booboo uttered each test sentence, and Experimenter 1 asked the child if Booboo was right. Then the child gave her response. Experimenter 1 gave feedback to Booboo that was in accordance with how the child had responded, as in the previous experiment. After both test sentences, Experimenter 1 flipped the card to see who would get to stamp and then asked the child the filler question. After the child responded, the appropriate player(s) stamped their paper(s), and we moved on to the next test trial. The entire experiment took around 20 min per child.

### Results

#### Coding

Children's responses were coded online by the second experimenter. Four out of the 55 videos were coded by a second experimenter offline, because coding did not happen online. Responses were coded as *yes*, or *no*. An additional 15 videos were coded offline by an additional coder to ensure reliability of the online coding. We found 97.9% agreement (Cohen's Kappa = 0.952).

#### Practice and filler accuracy

This experiment included an extensive training and practice section. There were four practice items. Children had to get all four in a row right to be included. We went through all the items either once (four items), twice (eight items), or three times (12 items). This means that children had a minimum of four practice items, and a maximum of 12. Ten out of the total of 55 children tested (18%) did not pass the practice after three rounds and were thus excluded from the rest of the experiment and analysis. Of the subjects for whom we recorded practice data, 26 went through the practice items once, 14 went through the practice items twice, and 3 went through the practice items three times. We find that overall performance was not different for the groups of children who did the practice items once or twice. The three children who did the practice items three times had lower overall performance (Table 6). This may be related to an overall difficulty for this group on this task, or it may be due to the fact that for these children the task took a longer amount of time, thus, they may have been more fatigued by the time they got to the test. For the additional children, the practice session was not recorded, due to a technical error.

**Table 6:** Experiment 2: accuracy by number of practice items.

**Number of rounds of practice items**	**% Appropriate responses on test items**
1	90
2	92
3	59
No data	85

The fillers were designed as a control to ensure that children were paying attention during the game as well as to keep them engaged. Once children were included after the practice phase, they rarely had any difficulty correctly saying who got to stamp after each card flip, and asking the child after each card flip was an extremely natural question during the game. If they incorrectly answered the filler, they were directed to try again.

#### Truth-value judgments

The results for each condition are shown in Table 7.

**Table 7:** Percent yes-responses by condition for Experiment 2.

**Conflict/No conflict (within subjects)**	**Outcome (within subjects)**	**Sentence (Froggy vs. Child desire)**	**Target**	**% Yes**
conflict	positive (child stamps)	“froggy wants”	No	13
		“you want”	Yes	80
conflict	negative (Froggy stamps)	“froggy wants”	Yes	74
		“you want”	No	15
no conflict	positive (Child & Froggy stamp)	“froggy wants”	Yes/No	40
		“you want”	Yes/No	41
no conflict	negative (No one stamps)	“froggy wants”	No	9
		“you want”	No	6

We used mixed-effect logit models (Bates and Sarkar, 2007) to analyze the present results. The reported models have random intercepts. We ran a mixed-effect logit model with “yes” as the dependent measure, with Conflict, Outcome, and Sentence as fixed effects, and Item and Subject as random effects. We tested for each of the two-way interactions, testing Target against a model dropping each of the fixed effects. We found a significant two-way interaction between interaction between Sentence and Outcome [${\text{X}}_{\left(1\right)}^{2}$ = 105.5, *p* < 0.0001], and a significant interaction between Sentence and Conflict [${\text{X}}_{\left(1\right)}^{2}$ = 7.59, *p* = 0.006]^8^. Children's responded differently based on whose desire was reported and whether that outcome was positive or negative, and their responses were much less influenced by the presence of a conflict.

**Figure 2:**
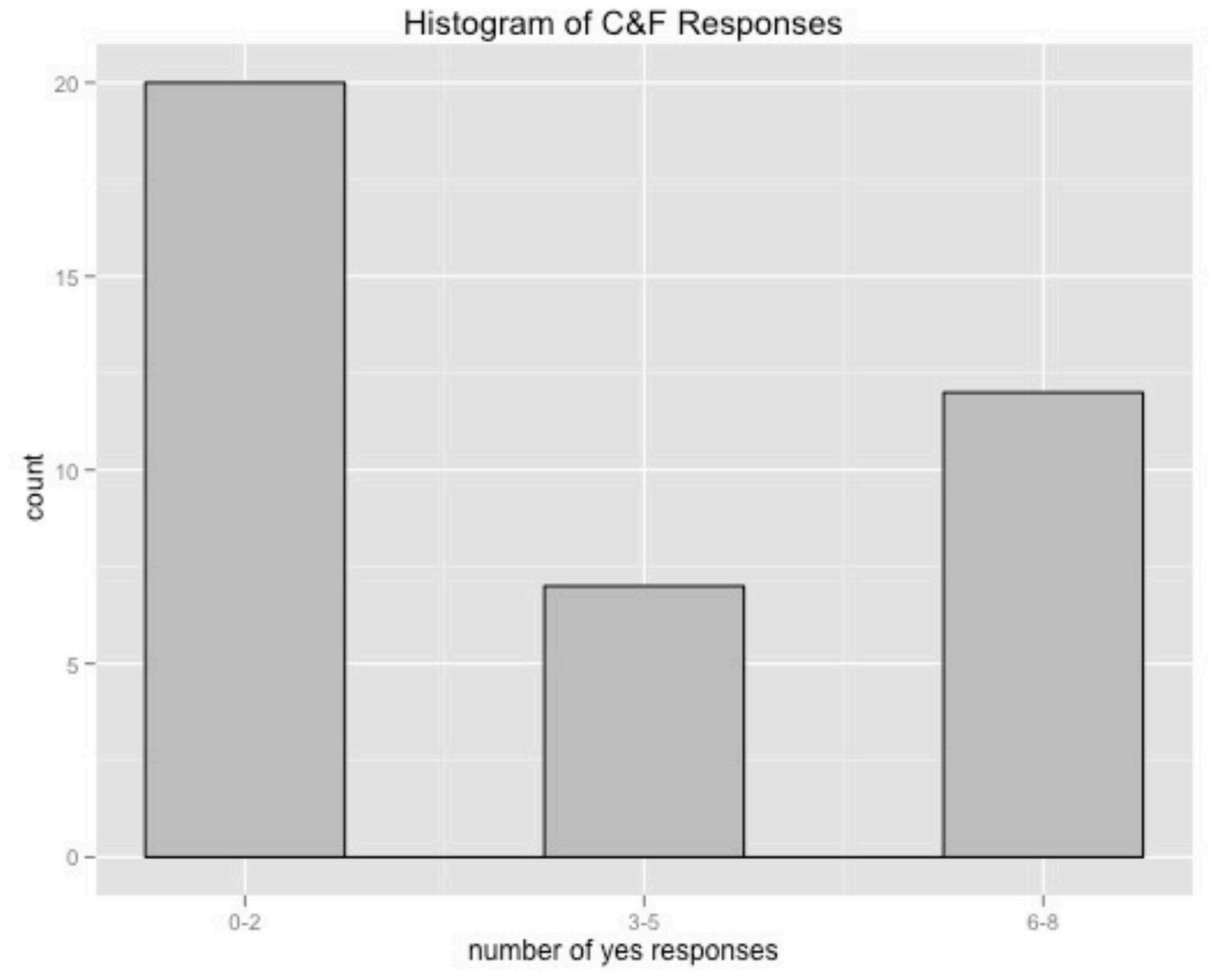
Histogram of responses to Child and Froggy condition.

Children were successful in the conflict cases, whether they were being asked about either positive or negative outcomes. In the negative no conflict condition, children were successful in saying that neither they nor Froggy wanted an outcome where no one got to stamp. In the positive no conflict condition, where both the child and Froggy got to stamp, some children played the game in a competitive way, responding that neither they nor Froggy wanted the outcome where both players got to stamp (*n* = 30; mean age = 3.6), while other participants were more charitable, responding that both they and Froggy wanted this outcome (*n* = 10; mean age = 3.8). The histogram in Figure 2 shows that children were normally choosing either a strategy of responding *yes* to this condition all the time, or *no* to this condition all the time. This indicates that they were not confused by this condition, but that different children simply differed in how competitive they chose to be with Froggy. This however, does not affect whether a conflict with someone else's desires impacts performance on interpreting *want*.

Our results indicate that 3-year-olds behave in an adult-like way in interpreting *want* even when they are asked to assess a character's desire that conflicts with their own, as long as they are given adequate training and opportunity to understand the rules of the “game” used to test this ability^9^. Importantly, when Froggy gets to stamp, the child does not get to stamp. So if the child's desire in the context of the game is to stamp, then a positive outcome for Froggy is a negative outcome for the child and *vice versa*. Thus, we can interpret children's correct interpretations of sentences about Froggy's desires as evidence of their ability to represent desires that conflict with their own.

One potential problem with this study is the possibility that the “desire” that we induced in the child in the game is not a real one. During the game, the child does not choose which color is linked to the opportunity for them to stamp, and the color-outcome pairs are constantly changing every four trials. The rotation of colors and outcomes was an important manipulation to ensure that the results were not affected by color biases, but it's possible that this manipulation had an effect on the strength of participants' desires. If the participants in this task did not have a strong or meaningful desire about the color of the card, this task could not successfully test conflicting desires, since children would not have to override their own desires about color. In order to control for this possibility, we ran a small sample (*n* = 8) on a slight modification of Experiment 2, designed to induce a more deeply rooted desire in children. In this manipulation, each child chose which color led to which outcome, and the color/outcome pairings did not change during the course of the game. This allowed the child to have a more deeply rooted desire, because they had a say over which color would mean a positive outcome for them, and this stayed consistent throughout the game. All other aspects of the task and analysis were identical to Experiment 2. We find the same pattern of performance in this control study, indicating that success in Experiment 2 is not due to the lack of a real desire in the participants.

### Discussion of experiment 2

Previous tests of *want* either did not require the child to evaluate desires that directly conflicted with their own, or the methodology was problematic and results were inconclusive. In order to fully understand children's early understanding of the verb *want*, it was necessary to further explore children's interpretations of *want* sentences under these conditions. Experiment 2 improved on previous methodology, and showed that 3-year-olds responded in adult-like fashion to *want* sentences used to report desires that conflicted with their own desires. This suggests that children's knowledge of the verb *want* and the underlying concept is robust at this age, and not disrupted by complex situations involving processing conflicting desires.

In order to truly test interpretation of *want* without the confound of interference from errors caused by lack of understanding of the game, it was critical to include only children who can show, outside the context of interpreting conflicting desires, that they understand its rules. However, it is possible that the children who were included in the test items of our task are children who are more advanced in other cognitive systems, such as executive function or memory. Given the evidence that children with higher executive function are better at tasks involving processing mental states (Leslie and Thaiss, 1992; Leslie and Roth, 1993; Leslie and Polizzi, 1998; Roth and Leslie, 1998; German and Leslie, 2000; Leslie et al., 2005; Rakoczy, 2010; Devine and Hughes, 2014; Fizke et al., 2014), it is possible that the sample of children who succeeded in learning the rules of our game were children who may have an independent advantage in understanding the test sentences. Furthermore, some evidence suggests that the ability to participate in a competitive game correlates with children's performance on the traditional false belief task (Priewasser et al., 2013). If so, the sample of children who were able to pass the training on this task may also have been more advanced in attributing mental states.

Experiment 2 shows that, as long as children are able to understand the rules of this game, they are successful in interpreting *want* sentences that report conflicting desires. We leave it to future research to probe further whether children that are unable to learn the game also have the ability to represent desires that conflict with their own.

## General discussion

The results from our experiments show that 3-year-olds understand *want* sentences in an adult-like way, even when they report counterfactual and conflicting desires, including desires that conflict with their own. This shows that by age three, children have an adult-like understanding of the verb *want*. It also shows that, even when measured by the most stringent standards, children are able to represent the subjective desires of others. Without an adult-like concept of desire, it is unclear how children could consistently correctly interpret *want* sentences reporting such conflicting desires.

These results also have implications for our understanding of children's understanding of the belief concept and the verb *think*. At the same age as we find success in interpreting *want*, children have notorious difficulty understanding *think* sentences when they report a false belief, that is, a belief that conflicts both with reality, and with their own beliefs. Our results suggest that children's difficulty with *think* cannot be explained solely as difficulty processing a report of a mental state which conflicts with reality or with their own mental state, and that the asymmetry observed in the acquisition of these two verbs is not due to differences in the ways in which these verbs have been tested.

Three possible sources for the asymmetry in children's understanding of *want* and *think* sentences can be found in the literature. First, it may reflect an asymmetry in the development of the *concepts* that these verbs express (cf. Perner, 1988, 1991; Tardif and Wellman, 2000; Perner et al., 2003, 2005; Perner and Ruffman, 2005; for an overview see Steglich-Petersen and Michael, 2015), perhaps because the concepts of belief and desire differ in complexity (see Flavell, 1988; Schwitzgebel, 1999, among others). According to this Conceptual Asymmetry hypothesis, the desire concept appears earlier than the belief concept, which awaits the development of a full *Theory of Mind*, around age 4, as evidenced by children's consistent failure at explicit false belief tasks before then (Wellman et al., 2001).

A number of recent studies with young infants however cast doubt on this hypothesis, and suggest that children show (implicit) understanding of belief very early, when tested through implicit measures (Woodward, 1998, 1999, 2003; Onishi and Baillargeon, 2005; Southgate et al., 2007; Kovács et al., 2010; Senju et al., 2011). Whether these infant results truly show belief understanding or not is a matter of active debate in the literature (see e.g., Apperly and Butterfill, 2009; Thoermer et al., 2012; Butterfill and Apperly, 2013; Heyes, 2014). But if young children lack the belief concept, or have difficulty accessing it in explicit tasks, such as tasks testing their comprehension of *think*, we expect them to have difficulty with *think* longer than with *want*.

Another possible explanation for children's ease with *want* and relative difficulty with *think* is that the differences in the acquisition trajectories are linguistic in nature. de Villiers (2005), for instance, argues for a syntactic Asymmetry in the acquisition of the syntax of tensed vs. untensed complements. This can explain why we see adult-like performance with *want* earlier than has been observed with *think*. Potentially problematic for this type of explanation, however, are languages like German. In German, *want* can also take a tensed sentential complement, but it still doesn't trigger the same errors as *think* sentences do, when the complement is false, which makes it doubtful that this specific piece of syntactic knowledge can entirely explain the asymmetry (cf. Perner et al., 2003). A more recent proposal from de Villiers assumes that the important feature of false complements must be semantic, having to do with how the different perspectives on a proposition are represented (de Villiers and de Villiers, 2009).

Alternatively, children's relative difficulty with *think* could be due to a Pragmatic Asymmetry in the kinds of pragmatic enrichments that these verbs trigger (Hacquard, 2014; Harrigan, 2015; Hacquard and Lidz, to appear). *Think* sentences can be used to make indirect assertions. Lewis and colleagues (Lewis et al., 2012, 2017; Lewis, 2013) provide evidence that such pragmatic uses might be responsible for children's tendency to reject *think* sentences when they report a false belief. *Want* sentences report preferences and, as such, are not routinely used to make indirect assertions; they thus do not lead children down the same pragmatic garden path as *think* sentences. As Lewis et al. (2017) showed that 3-year-olds were able to reject false *think* sentences that report a false belief, our own inclination is to lean toward this Pragmatic Asymmetry hypothesis (see Hacquard and Lidz, to appear for further elaboration).

## Conclusion

The two studies presented in this paper have probed 3-year-olds' knowledge of the verb *want* in contexts in which it is used to report conflicting or counterfactual desires. This was critical to get at children's knowledge of this verb, especially given the observed difficulty children of the same age have in interpreting *think* in equivalently complex situations. We find that 3-year-olds are adult-like in interpreting *want* sentences, even when they are used to report desires that conflict with reality, or with other desires, including their own.

## Ethics statement

The studies were carried out in accordance with the recommendations of the Institutional Review Board at the University of Maryland, College Park with written informed consent from the parents of all subjects. Parents of subjects gave written informed consent in accordance with the Declaration of Helsinki. The protocol was approved by the Institutional Review Board at the University of Maryland.

## Author contributions

KH: Experimental design, experimental materials, collecting, analyzing data, writing; JL and VH: Experimental design, analyzing data, writing.

### Conflict of interest statement

The authors declare that the research was conducted in the absence of any commercial or financial relationships that could be construed as a potential conflict of interest.

## Footnotes

^1^*Glauben* is traditionally translated as *believe*, but was glossed as *think* in Perner et al. (2003). Though there may be subtle meaning and usage differences between *think* and *believe*, both verbs are used to report beliefs that can be true or false.

^2^German *wollen* (*want*) also allows nonfinite complements when the implicit subject of the embedded clause is the same as the *wanter* [e.g., “*Andy will schlaffen” (“Andy wants to sleep”)]*.

^3^*Think* sentences in German can receive a future-oriented interpretation, but this requires an adverb, such as “*later*.” *Want* sentences get a future-oriented interpretation without an adverb by default.

^4^Because of the relatively high number of children excluded from this task, we did additional analyses to ensure that the conclusions drawn from the data reflect the general population of 3-year-olds. If we include the additional 15 children who had originally been excluded due to *yes* or *no* response biases, we find the same significant main effect of Truth and no other main effects or interactions.

^5^An anonymous reviewer points out that children's behavior is also consistent with children interpreting *want* as *tell* in these stories.

^6^An anonymous reviewer points out that both desired situations—Megan staying in the cart, and Megan getting cereal—are objectively desirable: the former makes Mom happy, the latter makes Megan happy. So there is reason for action to make both desirable situations happen. In the terms of Priewasser et al. (2013), the desires are incompatible (both can't be realized), but not contradictory (they can both be desirable). Hence these results may not be at odds with children being teleologists, in terms of the actions they expect of others. While this may be true, our goal here is to show that children understand *want* sentences which *describe* desires that conflict, but we can't tell how they use this information in reasoning about actions.

^7^The brown card was occasionally named by the child as a different color, e.g., *tan*. In these cases we adapted the name of the color based on what the child said. Children had no difficulty with any other colors used in the game.

^8^We also ran the same analyses including the children who were excluded due to *yes-* or *no-*biases. We find the same significant interaction between sentence and Outcome and a marginally significant interaction between sentence and conflict.

^9^An anonymous reviewer points out that children's behavior is also consistent with children interpreting *want* as *need* in this experiment.
