# Supplementary material for: Three-Year-Olds' Understanding of Desire Reports Is Robust to Conflict
Source: Front Psychol. 2018 Feb 19;9:119. doi: 10.3389/fpsyg.2018.00119 (PMC5826074; doi:10.3389/fpsyg.2018.00119)
Supplement: Supplementary file 1 [file DataSheet1.pdf]

Appendix -- Experiment 1 Materials

Practice Items:

Practice 1:

Story: *Stacey is at home, making a sandwich for her mom.*

Test Sentence: *Stacey is making a sandwich.*

Practice 2:

Story: *It is so hot outside, Alan is swimming at the pool.*

Test sentence: *Alan is playing on the playground.*

Story 1: Bedtime

Conflict/Switch:

Amy is in her room playing with toys. Amy's mom comes in and says "Amy, come downstairs, we have company!" Amy says "OK, mom!" Amy's mom leaves, and Amy says to herself, "I know my mom asked me to go downstairs, but I'm having too much fun playing, I'm going to stay here!" And she keeps playing with toys in her room.

No Conflict/Stay

Amy is in her room playing with toys. Amy's mom comes in and says "Amy, come downstairs, we have company!" Amy says "OK, mom!" Amy's mom leaves, and Amy says to herself, "I'd like to keep playing with my toys, but my mom asked me to come downstairs, so I'd better do it!" And she starts to go downstairs.

False filler: *Amy's dad came up to her room to talk to her.*

Test sentence: *Amy's mom wants her to be going downstairs right now.*

Story 2: Picking up from School

Conflict/Switch

Jimmy is at school reading with his friend. Dad comes to pick Jimmy up from school. Dad says, "It's time to go home! I'm going to go get your brother from his classroom, go outside and play and wait for me." And Jimmy says "OK, I will." Dad leaves, and Jimmy says to himself "I know my dad said to go outside and play and wait, but I'm having too much fun reading!" And he stays with his friend.

No Conflict/Stay

Jimmy is at school reading with his friend. Dad comes to pick Jimmy up from school. Dad says, "It's time to go home! I'm going to go get your brother from his classroom, go outside and play and wait for me." And Jimmy says "OK, I will." Dad leaves, and Jimmy says to himself "I'm having so much fun with my friend, I'd like to keep reading, but my dad said I have to go outside and wait, so I'd better do that" And Jimmy goes outside to play and wait.

True filler: *Jimmy's dad came to pick him up from school.*

False filler: *Jimmy's mom came to pick him up from school.*

Test sentence: *Jimmy's dad wants him to be outside playing right now.*

Story 3: At the doctor's office

Conflict/Switch

Jeffrey is in the waiting room at the doctor's office. While he waits for the nurse to come in, he plays with a stethoscope that he sees in the waiting room. The nurse comes in. She says, "Hi, Jeffrey, I'm going to come and take your temperature, but I have to go get my thermometer. Please put down that stethoscope and play with these blocks until I come back" and Jeffrey says "OK!" While the nurse is gone, Jeffrey says to himself, "I know the nurse said I should play with the blocks instead, but I'm having too much fun playing with the stethoscope!" And he keeps playing with the stethoscope.

No Conflict/Stay

Jeffrey is in the waiting room at the doctor's office. While he waits for the nurse to come in, he plays with a stethoscope that he sees in the waiting room. The nurse comes in. She says, "Hi, Jeffrey, I'm going to come and take your temperature, but I have to go get my thermometer. Please put down that stethoscope and play with these blocks until I come back" and Jeffrey says "OK!" While the nurse is gone, Jeffrey says to himself, "I'd like to keep playing with this stethoscope, but the nurse said I should play with these blocks instead, so I'd better do that." And he starts to play with the blocks.

True filler: *The nurse came into the waiting room to talk to Jeffrey.*

False filler: *The doctor came into the waiting room to talk to Jeffrey.*

Test sentence: *The nurse wants Jeffrey to be playing with the stethoscope right now.*

Story 4: In the kitchen

Conflict/Switch

Maggie is helping her sister bake cookies in the kitchen. Maggie is stirring a pot on the stove. Maggie's sister realizes she has to run to the store to get chocolate chips because they are out. She

says, "Maggie, that's enough stirring, could you go upstairs and play while I run to the store?" and Maggie says "OK!" When her sister leaves, Maggie says to herself "I know my sister told me to go upstairs and play, but I really like stirring this pot!" And she keeps stirring the pot anyway.

No Conflict/Stay

Maggie is helping her sister bake cookies in the kitchen. Maggie is stirring a pot on the stove. Maggie's sister realizes she has to run to the store to get chocolate chips because they are out. She says, "Maggie, that's enough stirring, could you go upstairs and play while I run to the store?" and Maggie says "OK!" When her sister leaves, Maggie says to herself "I really like stirring this pot, but my sister said I should go upstairs and play, so I'm going to!" And she goes upstairs to play.

True filler: *Maggie is baking cookies with her sister.*

False filler: *Maggie is baking cookies with her brother.*

Test sentence: *Maggie's sister wants her to be stirring the pot right now.*

Story 5: At School

Conflict/Stay

Alex is at school, playing dress-up. His teacher calls out to the class, "OK, everyone! I have to run next door, stay at the play stations you're in until I get back!" Alex says "OK!" Alex says to himself, "I know my teacher said I have to stay in the dress-up corner, but I'd like to go color, so I will!" And he goes over to color.

No Conflict/Switch

Alex is at school, playing dress-up. His teacher calls out to the class, "OK, everyone! I have to run next door, stay at the play stations you're in until I get back!" Alex says "OK!" Alex says to himself, "I'd like to go color, but my teacher said I have to stay here in the dress-up corner, so I will!" And he stays in the dress-up corner.

True filler: *Alex is playing at school.*

False filler: *Alex is playing at home.*

Test sentence: *The teacher wants Alex to be playing dress-up right now.*

Story 6: At the office

Conflict/Stay

Jeremy's teacher sent him to the office to pick something up for her from the principal. Jeremy is waiting for the principal and looking at a book. The secretary comes over to Jeremy. She says, "the principal is talking to someone right now, sit here and keep looking at that book until he's ready for you." And Jeremy says "OK!" When the secretary turns away, Jeremy says to himself "I know the secretary said that I should keep looking at this book, but I'd like to see what's happening in the principal's office." And he goes over and peeks into the principal's office.

No Conflict/Switch

Jeremy's teacher sent him to the office to pick something up for her from the principal. Jeremy is waiting for the principal and looking at a book. The secretary comes over to Jeremy. She says, "the principal is talking to someone right now, sit here and keep looking at that book until he's ready for you." And Jeremy says "OK!" When the secretary turns away, Jeremy says to himself "I'd really like to peek in the principal's office right now, but the secretary said I should keep looking at this book, so I will." And he keeps looking at the book.

True filler: *Jeremy is waiting in the principal’s office.*

False filler: *Jeremy is waiting in the doctor’s office.*

Test sentence: *The secretary wants Jeremy to be looking at a book right now.*

Story 7: Grocery shopping

Conflict/Stay

Megan is at the grocery store with her mom, and she is sitting in the cart while her mom shops. Megan’s mom says, “Megan, I have to run and get something in the next aisle, stay right there in the cart until I get back,” and Megan says “No problem, mom!” Megan says to herself, “I know my mom said to stay in the cart, but I’d like to get out and go get some cereal, so I will.” And she climbs out of the cart to go get some cereal.

No Conflict/Switch

Megan is at the grocery store with her mom, and she is sitting in the cart while her mom shops. Megan’s mom says, “Megan, I have to run and get something in the next aisle, stay right there in the cart until I get back,” and Megan says “No problem, mom!” Megan says to herself, “I’d like to get out of the cart and go get some cereal, but my mom said to stay in the cart, so I will.” And she stays in the cart.

True filler: *Megan is at the grocery store with her mom.*

False filler: *Megan is at the grocery store with her dad.*

Test sentence: *Megan’s mom wants her to be getting cereal right now.*

Story 8: After dinner

Conflict/Stay

After dinner, Doug is playing a computer game while his parents clean up from dinner. His dad comes in and says, "Doug, it's going to be time for bed soon, just play your game for awhile while we clean up from dinner." and Doug says "OK, dad!" Doug says to himself, "I know my dad said I should keep playing my game, but I would really like to go outside and play, so I will." And he goes outside to play.

No Conflict/Switch

After dinner, Doug is playing a computer game while his parents clean up from dinner. His dad comes in and says, "Doug, it's going to be time for bed soon, just play your game for awhile while we clean up from dinner." and Doug says "OK, dad!" Doug says to himself, "I'd really like to go outside and play right now, but my dad said I should keep playing my computer game because it's almost time for bed." And he keeps playing his computer game.

True filler: *Doug just finished eating dinner.*

False filler: *Doug just finished eating breakfast.*

Test sentence: *Doug's dad wants him to be outside playing right now.*
